# A bifunctional superconducting cell as flux qubit and neuron

**DOI:** 10.3762/bjnano.14.92

**Published:** 2023-11-21

**Authors:** Dmitrii S Pashin, Pavel V Pikunov, Marina V Bastrakova, Andrey E Schegolev, Nikolay V Klenov, Igor I Soloviev

**Affiliations:** 1 Faculty of Physics, Lobachevsky State University of Nizhni Novgorod, 603022 Nizhny Novgorod, Russia; 2 Russian Quantum Center, 143025 Skolkovo, Moscow, Russia; 3 Skobeltsyn Institute of Nuclear Physics, Lomonosov Moscow State University, 119991 Moscow, Russia; 4 Moscow Technical University of Communication and Informatics (MTUCI), 111024 Moscow, Russia; 5 Faculty of Physics, Lomonosov Moscow State University, 119991 Moscow, Russia; 6 National University of Science and Technology MISIS, 119049 Moscow, Russia

**Keywords:** adiabatic logic cell, flux qubit, Josephson junctio, quantum neuron, quantum parametron, superconducting quantum computers, superconducting quantum interferometer

## Abstract

Josephson digital or analog ancillary circuits are an essential part of a large number of modern quantum processors. The natural candidate for the basis of tuning, coupling, and neromorphic co-processing elements for processors based on flux qubits is the adiabatic (reversible) superconducting logic cell. Using the simplest implementation of such a cell as an example, we have investigated the conditions under which it can optionally operate as an auxiliary qubit while maintaining its “classical” neural functionality. The performance and temperature regime estimates obtained confirm the possibility of practical use of a single-contact inductively shunted interferometer in a quantum mode in adjustment circuits for q-processors.

## Introduction

Superconducting interferometers are widely used both as flux qubits and as a part of the peripherals in various implementations of quantum computers [1–10]. In particular, the D-Wave 2000Q quantum computer, released in 2017, operates on the principle of quantum annealing and contains a superconducting chip with 128,472 Josephson junctions, 75 percent of which were dedicated to superconducting digital electronics for controlling the processor and reading out the result. The rest was used either for qubit junctions or for interconnects that allow for programmable interaction between qubits. The Pegasus P16 superconducting chip of the Advantage QA system, released in 2020, contained 1,030,000 Josephson junctions, of which only 40,484 were used for interconnects, and 5,640 Josephson structures were part of the qubits. In this context, the desire of designers to find additional uses for multiple “auxiliary” interferometers on a chip is understandable.

The least “noisy” option for building the bulk of such quantum computing systems is based on the concepts of adiabatic superconducting logic (ASL), which can operate at millikelvin temperatures with zeptojoule energy efficiency [11–17]. In addition, the basic cells of adiabatic superconducting circuits can be used as a part of neuromorphic co-processors [18–23] working in conjunction with quantum computing systems [24–32].

Furthermore, a natural extension of current progress would be the use of “quantum” degrees of freedom for adiabatic superconducting circuits, which share many similarities with qubits in terms of their representation of information via magnetic flux. From a formal point of view, the system under consideration is a superconducting circuit in a quantum state, transforming the input magnetic flux Φ_in_ into an output magnetic flux Φ_out_ according to a specific (e.g., sigmoidal) function Φ_out_ = *f*(Φ_in_) [33–34]. If we only want to use the circuit in the “classical” neuromorphic mode, the transfer characteristic should be such that small fluctuations at the input do not produce a noticeable response, but above a certain threshold, any signal at the input produces a fixed magnetic flux at the output. Also, if it were possible to adapt the ASL cell in a perceptron to process the signal from a qubit representing its quantum state restructuring the one’s spectrum in a certain way, we would have an auxiliary qubit that neither requires a highly stable reference oscillator nor a mixer to drive it. Of course, such a bifunctional cell as a qubit is not ideal; however, in some situations the gain in the “payload” on the chip may be more important.

**Figure 1:**
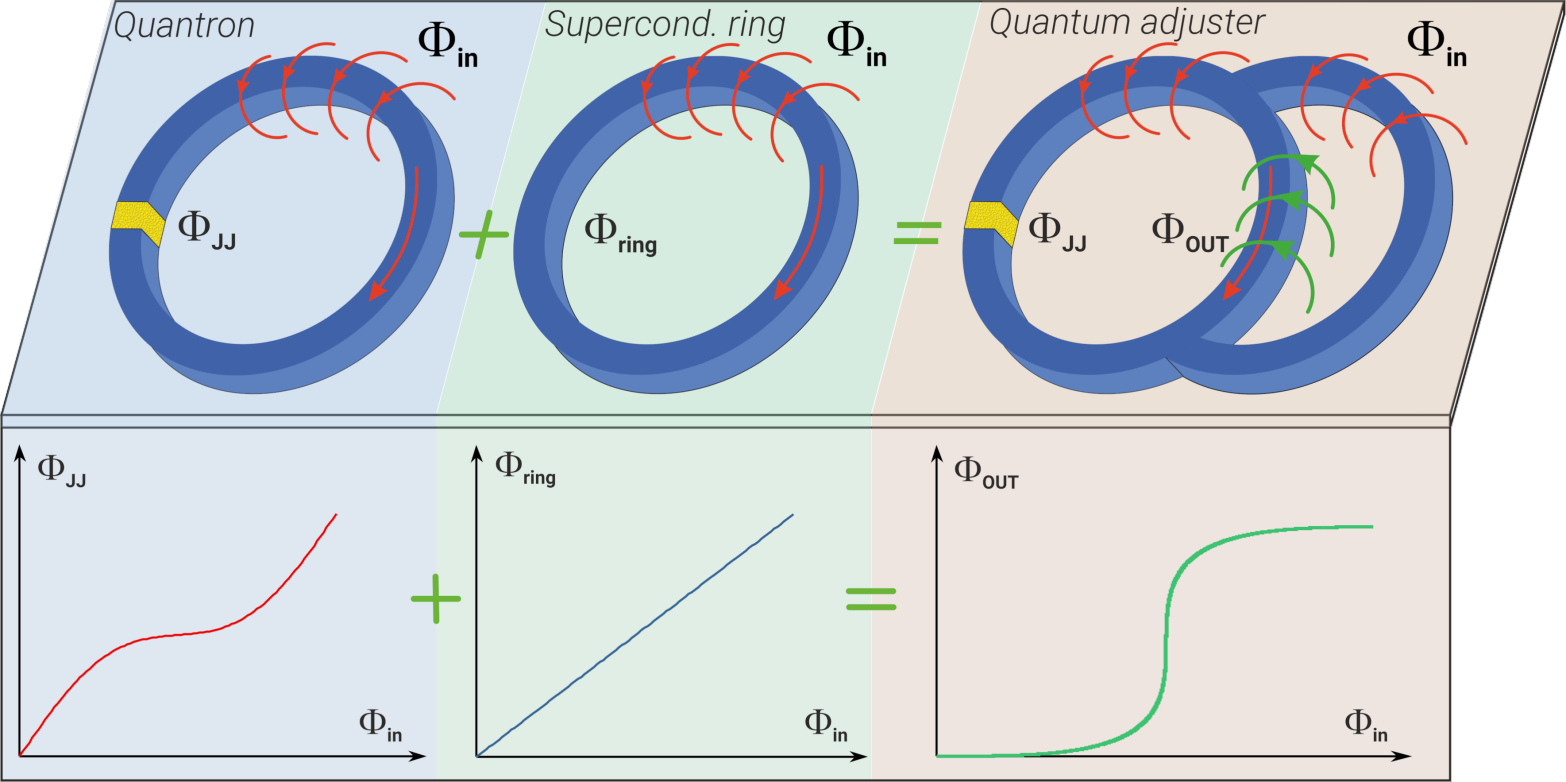
The idea behind the creation of the bifunctional cell. The combination of a quantum interferometer (quantron) and a simple superconducting ring leads to the emergence of a parametron with a sigma-like transfer function. Φ_in_(*t*) and Φ_out_(*t*) are the normalised fluxes at the input and output of the circuit; Φ_JJ_(*t*) and Φ_ring_(*t*) correspond to the superconducting phase drops at Josephson heterostructure and superconducting inductor, respectively. Nota bene, such a transfer characteristic is a good activation function for a neuron in a perceptron-type network, suitable for primary signal processing for quantum computing systems.

Apparently, the simplest superconducting circuit with a nonlinear flux-to-flux transformation in the classical regime is a single-contact interferometer, as depicted in the left part of Figure 1. However, the typical form of the function *f* for such an element does not meet the aforementioned requirements. In the classical mode, it can be demonstrated [28] that the desired form of *f*(Φ_in_) can be achieved by adding an inductance with a specially chosen linear flux-to-flux transformation to the interferometer, as illustrated in the right part of Figure 1. At zero temperature and under quasi-adiabatic changes in the circuit’s inputs, the desired transformation (now for average values) will occur even in the quantum regime, when the spectrum of eigenvalues of the system’s Hamiltonian operator is discrete. Nevertheless, it raises the question of how the proposed adjustment circuit will operate at finite (millikelvin) temperatures in the quantum regime and under the influence of relatively fast magnetic field control pulses? Will the tuning, coupling, and neromorphic co-processing circuits acquire new useful properties in the quantum regime?

This article is devoted to the search for answers to these questions. Hence, below we explore the quantum dynamics of observables in superconducting interferometers, discuss the implications for quantum computing, and the challenges that remain to be addressed. In addition, we note the potential for utilising the findings to develop components of neuromorphic co-processors that collaborate with quantum computing systems. We will refer to the corresponding cell (a single-contact interferometer shunted by an inductance as depicted in Figure 1) further in the text as the “parametron”.

## Model of the Proposed Bifunctional Cell

In this and subsequent sections, we consider a parametric quantron (parametron) under the influence of unipolar pulses of external magnetic flux. It should be noted that this system has proven to be a basic element of neural networks such as the perceptron with a sigmoidal input-to-output transformation function (sigma-neuron). Preliminary calculations have shown that under certain conditions, such a neuron can operate successfully in both classical and quantum modes [24,26,28,30]. The energy of the system in the Hamiltonian formalism can be expressed as follows:


[1]

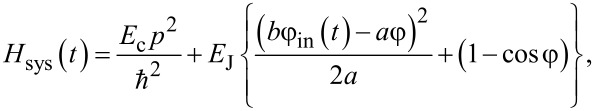



where the coefficients *a* and *b* are defined by the following expressions [28,30]:


[2]

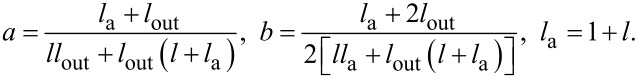



Here, *l* is the normalised inductance of the quantron part of the sigma-interferometer (*l* = 2π*LI*_c_/Φ_0_), *l*_a_ is the additional linear inductance, and *l*_out_ is the output inductance (*l*_a_ and *l*_out_ are normalised in the same manner as *l*). Here and further, we use magnetic fluxes normalised by the quantum value, Φ_0_.

To investigate the flux-to-flux transfer characteristics of such a system, it is convenient to interpret its evolution as the movement of a particle with “mass” *M* = 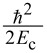 and “momentum” *p* = 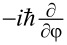 in the potential profile defined by the second term in Equation 1, wherein the effective coordinate is a phase of the Josephson junction, φ. The quantities *E*_c_ = 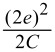 and *E*_J_ = 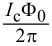 are the capacitive and the Josephson energy, respectively, determined by critical current *I*_c_ and the capacity *C* of the Josephson junction. A typical example of “flux-based” system state management is provided by the dynamically varying input magnetic flux:


[3]

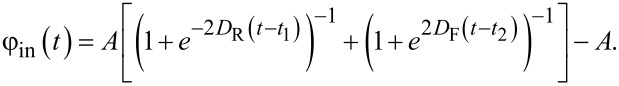



This flux pulse is characterised by the level *A*, rise/fall rate of the signal *D*_R/F_, and the characteristic times *t*_1_ and *t*_2_ = 3*t*_1_ responsible for the rise and fall periods of the input magnetic flux. It is assumed that the time is given in units of 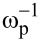, where 
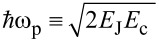
.

In the framework of the adiabatic approach, one can numerically find “instantaneous” energy levels *E*_n_(*t*) and “instantaneous” eigenfunctions |ψ_n_(*t*)⟩ of the system solving the stationary Schrödinger equation using the finite difference method [35]:


[4]

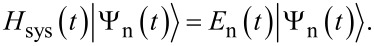



If at each moment in time, the state energy *E*_n_(*t*) is much smaller than the distance between energy levels in the system, then the adiabatic approximation is valid and, therefore, transitions between instantaneous eigenstates can be neglected. Mathematically this condition can be expressed as:


[5]

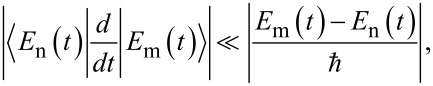



which just defines the standard Landau–Zener problem [36–38]. If the adiabatic approximation is violated, for example, when the energy levels *E*_n_(*t*) and *E*_m_(*t*) converge (anticrossing), Landau–Zener transitions occur. The rate of these transitions is controlled by the form of the external influence. At moments of level convergence for short periods τ_LZ_, the phases of the wave functions change significantly, leading to strong fluctuations of the level populations in the system, and can lead to quasi-random dynamics in the parametron. In addition, Landau–Zener interference has become a tool to access the multilevel structure of these artificial atoms [39–42]. It is also used to obtain information about the connection of the qubits with a noisy environment and to form dissipative stable entanglements in quantum tomography protocols [43–45]. Let us further consider the limitations that such non-adiabatic effects impose on the potential use of the proposed cell as a neuromorphic, coupling, and tuning element in quantum computing systems. At the same time, we will also gain an understanding of the possibilities for controlling the population of levels in the simplest implementation of an adiabatic superconducting logic cell when used as an auxiliary qubit.

## A Bifunctional Superconducting Cell as a Controllable Flux Qubit

**Figure 2:**
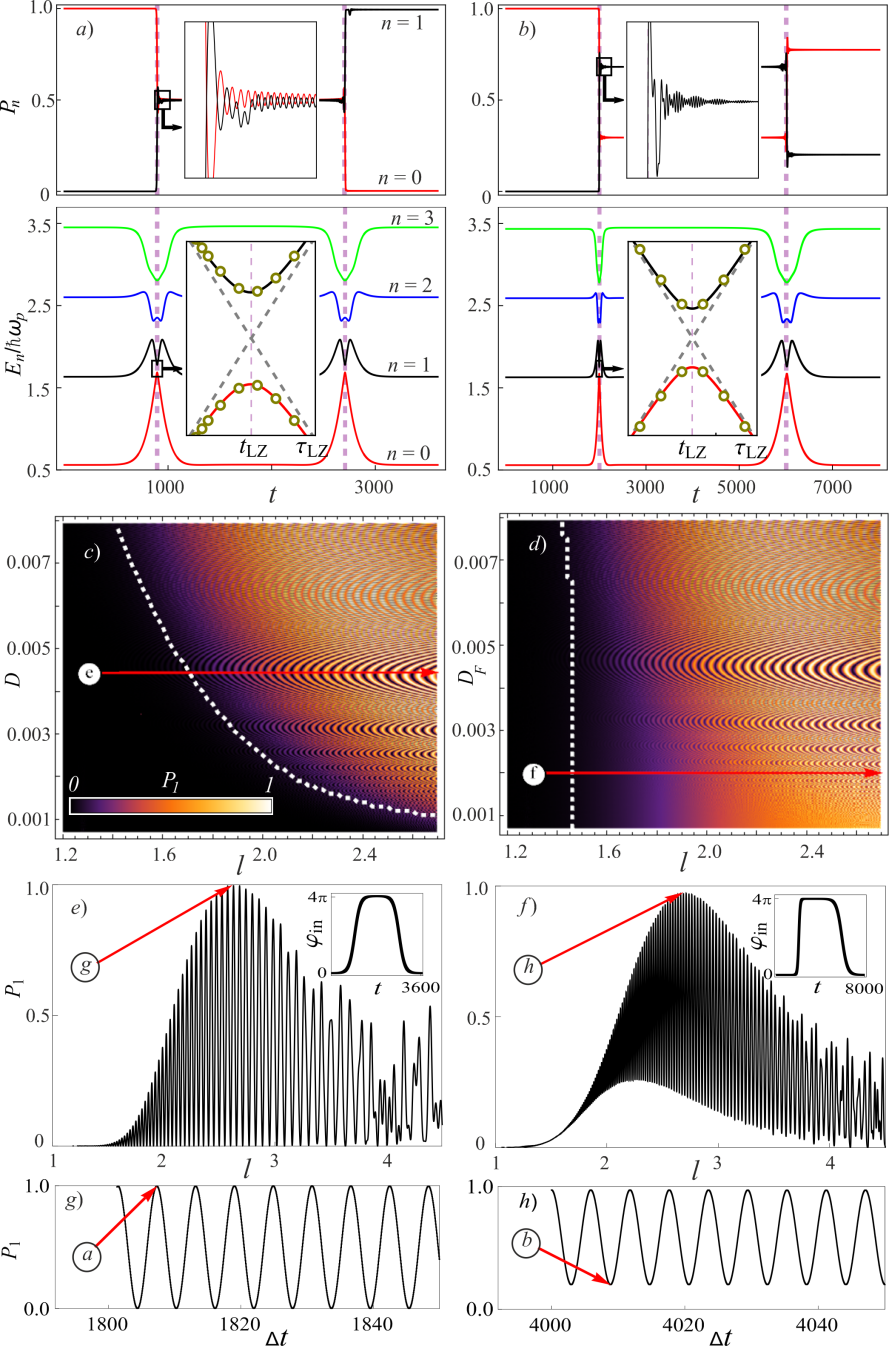
(a, b) Time-dependence of the populations of ground state, *P*_0_(*t*), (black curve) and first excited state, *P*_1_(*t*), (red curve). Additionally, the four lowest states *E*_i_(*t*) of the quantum interferometer are also demonstrated for (a) *l* = 2.63 and *D* = 0.0044 and (b) *l* = 2.69, *D*_R_ = 0.008 and *D*_F_ = 0.002. Diabatic levels for *E*_0_*_,_*_1_ are shown in the insert by the dashed line, and analytical estimations in the two-level approximation (from Equation 7) are shown by dots. (c, d) Interference population map for the first excited state for various values of the inductance *l* and rates of change of the control field fronts *D*_R/F_. The white dashed line denotes the violation boundary of the adiabatic approximation according to Equation 8 with accuracy equal to *P*_LZ_ *>* 1%. For *D* = 0.0044 (e) and *D*_R_ = 0.008 and *D*_F_ = 0.002 (f), cross sections of probabilities *P*_1_(*l*) are given, which are marked with red arrows in (c, d). (g, h) Population of the excited state as a function of Δ*t* = *t*_2_ − *t*_1_ with fixed values of (a) *l* = 2.63 and (b) *l* = 2.69 at the end of external influence. The plots in (a, c, e, g) were calculated for a symmetrical φ_in_(*t*), while the plots in (b, d, f, h) were calculated for an asymmetrical input flux. The parameters of the system were *l*_a_ = 1 + *l*, *l*_out_ = 0.1, *E*_J_ = 2*E*_c_, and *t*_1_ = 3*t*_2_.

The state dynamics of the considered system equation (Equation 1) are primarily defined by features of the controlling field equation (Equation 3), as well as by the values of the inductances. We have considered two cases of external field influence to the system, namely (i) when the controlling field has symmetrical rise/fall fronts, *D*_R_ = *D*_F_ = *D*, and (ii) when it does not, *D*_R_ ≠ *D*_F_. It is assumed that at the initial time, the system is initialised to the ground state, that is, localised at the level with energy *E*_0_. The evolution of energy level population and instantaneous energy levels was numerically calculated for the *n* = 10 lowest energy levels of the quantum interferometer. As shown in Figure 2a,b, during rise and fall of the field, the instantaneous energy levels are getting closer, and the anticrossing effect is observed. For the ground and first-excited states, characteristic times of anticrossing correspond to τ_LZ_, when the adiabatic condition (Equation 5) is violated and a non-zero probability of Landau–Zener tunneling between these energy levels emerges. As long as the leakage to upper states (for *n >* 2) during such transitions is less than |*P*_1_ − *P*_0_| − *P_n_*_≥2_ ≪ |*P*_1_ − *P*_0_|, the two-levels approximation (*n* = 2) can be applied for analytical estimations of Landau–Zener transition probabilities [39,42]. Within this approximation, the system can be approximated by the following Hamiltonian:


[6]

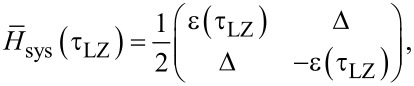



where Δ = *E*_1_(*t*_LZ_) − *E*_0_(*t*_LZ_) determines the distance between energy levels at the moment of their closest convergence, and ε(τ_LZ_) determines the type of levels anticrossing. The instantaneous energy levels of the ground and excited states can then be written as *E*_0_*_,_*_1_(τ_LZ_) = 
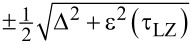
.

Let us estimate the Landau–Zener transition probability at the moment of the first levels’ convergence, which corresponds to the time *t*_LZ_ for diabatic dynamics (when Δ → 0) of level crossing (dashed lines in inserts in Figure 2a,b). As clearly seen from the simulation, the anticrossing effect occurs on small time scales near the moment of convergence *t*_LZ_. This allows us to use a linear approximation on time ε(*t*_LZ_ + τ_LZ_) ≈ ε′(*t*_LZ_)·τ_LZ_ and write the Hamiltonian of the system as:


[9]

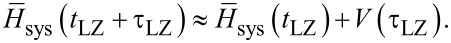



We assume that *V*(τ_LZ_) = 
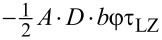
 is small on the scale of the Landau–Zener transition time. This allows us to use the perturbation theory to estimate the value of ε′(*t*_LZ_). In the moment of anticrossing, instantaneous energy levels *E*_0_ and *E*_1_ reach their extremum. Therefore, it is necessary to take into consideration the second order of perturbation theory for an analytical estimation of the convergence value:


[10]

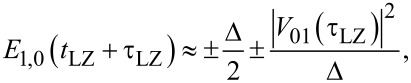



where *V*_01_(τ_LZ_) ≡ ⟨ψ_0_(*t*_LZ_)|*V*(τ_LZ_)|ψ_1_(*t*_LZ_)⟩. Finally, from Equation 10 the difference between the energy levels is


[11]

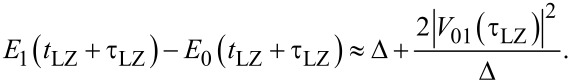



Expanding the row up to the second order, we can get the difference between the levels:


[7]

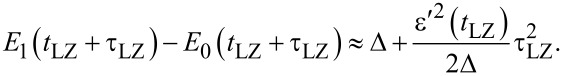



Then, from Equation 11 and Equation 7, we obtain:


[12]

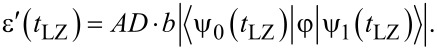



The dots in the insets to Figure 2a,b show the behaviour of the adiabatic energy levels at anticrossing τ_LZ_. The estimates obtained in the framework of the two-level model are in good agreement with the numerical calculations (solid lines in Figure 2a,b) of the Schrödinger time-dependent equation by the Crank–Nicolson method [46] based on the Magnus decomposition for the evolutionary operator up to the fourth order. This agreement indicates the correctness of the approximations used for the estimation. Based on the resulting expression for the linear coefficient expression (Equation 12) for ε(*t*_LZ_ + τ_LZ_), we use the well-known formula for calculating the probability of Landau–Zener transitions [39,42] with a single convergence of the levels:


[8]

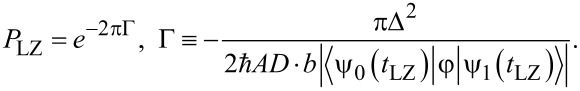



Using the obtained formula (Equation 8), we estimate the limit of occurrence of Landau–Zener transitions for different parameters of the control field and inductances in the circuit. To do this, we calculated the interference probability maps of the populations of the first excited level for typical quantum well inductances *l* and different parameters *D*_R,F_ for symmetric (Figure 2c) and asymmetric (Figure 2d) external control fields at the time corresponding to the end of the external influence. Bright areas in Figure 2c,d correspond to regions where there is a non-zero probability of quantum-coherent Landau–Zener tunneling, and black areas correspond to the adiabatic control of the system. According to the expressions in Equation 8, the white dashed line in Figure 2c denotes the limit of the transition probability from the ground to the excited state *P*_LZ_ *<* 0.01. This estimate is important for evaluating the functioning of this circuit in adiabatic quantum neural networks, where there are strict requirements for the absence of excitation from the initial state for the implementation of sigmoidal activation functions [30].

We can see from Figure 2e that for the symmetric control field for given *D*_R,F_, there are ranges of inductance values *l* where we can control the populations of levels by external influence using the Landau–Zener tunneling effect. In other words, in this parameter range we can, if necessary, control the state of the simplest adiabatic cell used as an auxiliary qubit. This parameter range is also important for the observation of quantum non-perturbative effects for the parametron that acts as a nonlinear adjuster, implementing the interaction between fluxonium type qubits [47–48]. In the case of an asymmetric control field, see Figure 2f, there is no complete transition between the *E*_0_ and *E*_1_ states in the system for a wide range of inductances, indicating the practical expediency of using a symmetric control influence. Another way of controlling the change in the level populations in the system is to control the phase difference between a pair of converging levels in the regions of increase (or decrease) in the external field, which of course depends on Δ*t* = *t*_2_ − *t*_1_. These dependencies are naturally periodic, as shown in Figure 2g,h, for the two cases of application of an external field.

The action time of the symmetric input flux to avoid Landau–Zener transitions is ∼100 ns for *l* = 2. The estimate was made with the characteristic parameters of a Josephson junction, that is, *I*_c_ = 50 nA and *C* = 6 fF. In contrast, it takes ∼30 ns for the transition from the ground state to the first excited state, as shown in Figure 2a. It can be assumed that the characteristic duration of the “NOT” operation will be of the same order of magnitude for the “flux control” of the tuning circuit cell used as an auxiliary qubit.

## A Model for Dissipative Effects in the Bifunctional Cell

Another important aspect to consider is the investigation of the impact of dissipative and temperature effects on the nonlinear dynamics of quantum interferometers. Quantum noise can result in the breakdown of coherence in the system and affect the operation of the parametron within coupling circuits and tuning schemes. In order to accurately describe these processes, we present the complete Hamiltonian of the system as:


[13]

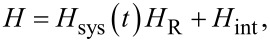



where *H*_sys_(*t*) is defined by Equation 1, and *H*_R_ is the energy of the thermal bosonic reservoir of the form:


[14]

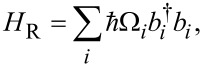



where Ω*_i_* is the frequency of the *i*-th bosonic mode, and 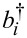 and *b_i_* are, respectively, creation and annihilation operators for the *i*-th bosonic mode. *H*_int_ is responsible for the interaction between the thermostat and the superconducting parametron. For the case of ohmic dissipation, this relationship is linear and can be written as:


[15]

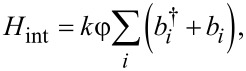



where *k* is a coupling constant.

Within the framework of the adiabatic approximation, we can form the density matrix of the system in the instantaneous basis |ψ*_n_*(*t*)⟩ as


[16]

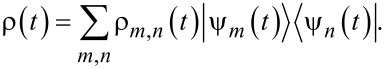



In the Born–Markov approximation, the dissipative dynamic of a quantum system is described by an adiabatic generalised equation for the density matrix [49]. In terms of the instantaneous basis in the Schrödinger representation, the dynamics of the parametron obey the Redfield equation:


[17]

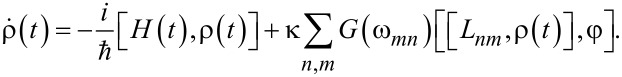



with 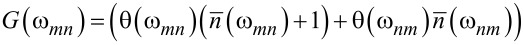 and *L_nm_* = |ψ*_n_*(*t*)⟩⟨ψ*_m_*(*t*)|⟨ψ*_n_*(*t*)|φ|ψ*_m_*(*t*)⟩. Here, we do not take into account the Lamb shift; 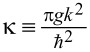 is the renormalised coupling constant, where *g* is the density of bosonic modes, θ is the Heaviside function, and 
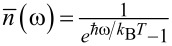
 is the Planck distribution. Note that we believe that the renormalised coupling constant does not depend on the frequency of the bosonic mode. We also used a numerical solution of the Redfield equation based on the Fock representation [50] using supercomputer modeling tools to obtain the results.

It should be noted that Equation 17 is valid under the standard adiabatic condition: *h*/δ^2^ ≪ 1, where 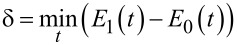 and 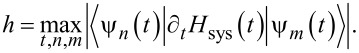 The ratio *h*/δ^2^ ≈ 0.08 for the characteristic parameters *l* = 2 and *D* = 0.001.

We will apply the described model to analyse the limitations on the operating temperature range when using the proposed parametron in coupling circuits and tuning schemes in quantum computing systems.

## A Bifunctional Superconducting Cell as an Adiabatic Neuron

The analysis conducted in section “A Bifunctional Superconducting Cell as a Controllable Flux Qubit” showed that Landau–Zener transitions significantly affect the dynamics of the system. Even in the case of adiabatic control, relaxation and thermal excitation processes can introduce additional difficulties that need to be considered when designing quantum interferometers and tuning circuits, adjusters, and neurons based on them. In particular, dissipative processes significantly affect the flux-to-flux transfer characteristics of such systems. In [30], we demonstrated that increasing the coupling coefficient of the interferometer with the reservoir suppresses oscillations of the mean flux value (generalised coordinate) caused by nonadiabatic interference effects. However, another important factor (in addition to relaxation) that influences the evolution of observable quantities for an interferometer is thermal fluctuations. It is known that the operating temperature, *T*, of quantum circuits with Josephson junctions is chosen much smaller than the characteristic temperature scale given by the distance between their ground and first excited energy levels:


[18]

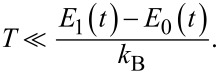



**Figure 3:**
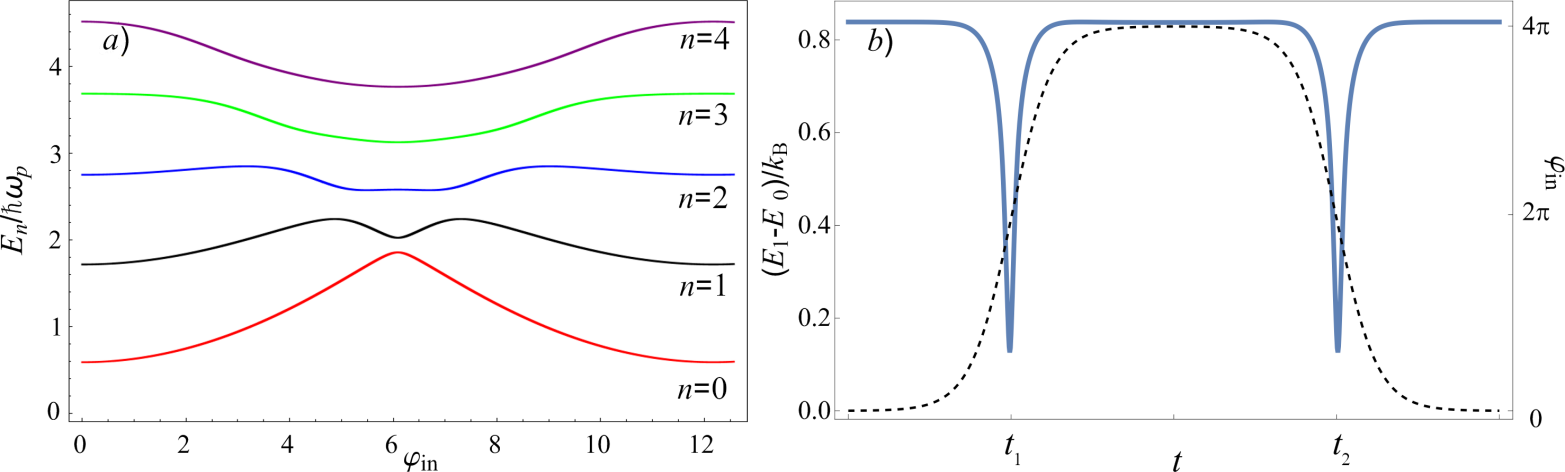
(a) The spectrum of the Hamiltonian (Equation 1) as a function of the external flux φ_in_(*t*) based on the numerical finite difference method [35]. (b) Blue solid line: temporal dependence of the distance between the ground state, *E*_0_(*t*), and the first excited state, *E*_1_(*t*), in the instantaneous basis of the parametron in quantum regime. Black dashed line: dependence of the input magnetic flux φ_in_ on time. The parameters of the circuit are *l* = 2, *D* = 0.001, *A* = 4π, *l*_a_ = 1 + *l*, *l*_out_ = 0.1, *E*_J_ = 2*E*_c_, *t*_1_ = 4000, and *t*_1_ = 3*t*_2_.

At the same time, the probability of reaching higher energy levels is proportional to


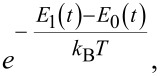


and the distance between the instantaneous energy levels depends on the applied external control field φ_in_(*t*), see Figure 3a. For example, for the parameters *l* = 2 and *D* = 0.001, corresponding to the adiabatic control region with symmetric magnetic flux (see Figure 2c), the energy gap between ground and first excited states attains its minimum value equal to 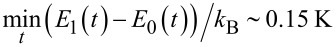 during increase and decrease of the external flux (the common form of the dependence is shown in Figure 3b). During these time intervals, the condition in Equation 18 may be violated, leading to transitions to higher energy levels. Therefore, an analysis of the parameter behaviour as a function of the working temperature is required to find operating modes where the probability of such thermal transitions is minimised.

We focused our attention on macroscopic observables in the parametron in the quantum regime, such as the transfer characteristic *i*_out_ = *f*(φ_in_). For the considered scheme shown in Figure 1, this dependence can be expressed through the following relation:


[19]

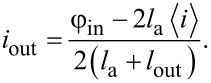



**Figure 4:**
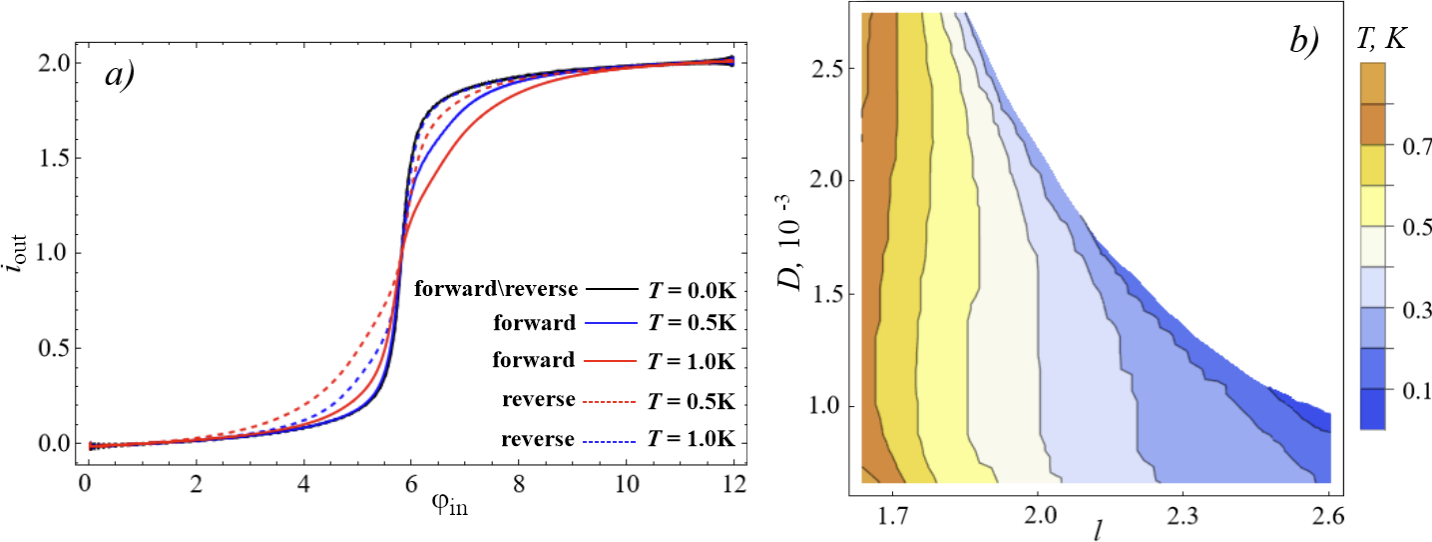
(a) Influence of temperature on the transfer characteristic of the parametron in the quantum regime. The solid lines represent the “forward” evolution of the system (φ_in_ = 0 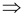 φ_in_ = *A*), and the dashed lines represent the “reverse” evolution (φ_in_ = *A* 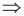 φ_in_ = 0). The black curve corresponds to the zero-temperature case, while the blue and red curves correspond to temperatures of 0.5 and 1 K, respectively. (b) The region of parameters where the transfer characteristic is close to a sigmoidal shape with a standard deviation of SD *<* 10^−4^. In the white area, even at zero temperature, the standard deviation is larger then SD = 10^−4^. The system parameters used in the simulation were *A* = 4π, a coupling coefficient with the thermostat determined by condition κ = 0.0025ω_p_, *l*_a_ = 1 + *l*, *l*_out_ = 0.1, *E*_J_ = 2*E*_c_, and *t*_1_ = 3*t*_2_.

Here, ⟨*i*⟩ = *b*φ_in_(*t*) − *a*⟨φ⟩ is the mean value of the current operator on the Josephson junction when the external flux changes relative to the mean phase of the contact ⟨φ⟩ = ⟨ψ(*t*)|φ|ψ(*t*)⟩. As shown in Figure 4a, the transfer characteristic of the parametron has a sigmoidal dependence. It is worth noting that this feature allows for the use of the proposed scheme in superconducting neural networks, such as perceptrons, integrated into hybrid quantum-neuromorphic computers. Moreover, the temperature affects the steepness of the sigmoid function. Even the manifestation of hysteresis in flux-to-flux transformations (when *i*_out_(φ_in_) during the increase of the external signal φ_in_ = 0 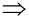 φ_in_ = *A*, solid lines in Figure 4a, does not coincide with the behaviour of the mean values during the decrease of the magnetic signal φ_in_ = *A* 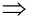 φ_in_ = 0, dashed lines in Figure 4a). We also emphasise that the sigmoidal transfer characteristic obtained is very useful for using the adiabatic cell in question as an auxiliary qubit. This feature of the system’s behaviour, together with the possibility of tuning the energy spectrum, makes it possible to minimise its parasitic “magnetic” influence on the environment.

Figure 4b presents the temperature map showing the maximum temperature at which the transfer characteristic of the parametron is sufficiently close to a sigmoid. To construct this map, we considered curves for which the standard deviation, *SD*, from the mathematical sigmoid did not exceed 10^−4^. The ordinate and abscissa axes correspond to the rise/fall rates of the applied flux and the normalised inductance of the cell, respectively. The calculations show that as the inductance of the parametron *l* and the performance of it increases, the requirements for system temperature control also increase, necessitating increasingly lower operating temperatures. For example, the dark blue region in Figure 4b is only suitable for *T* ∼ 0.1 K. Note that for the parameters used and a Josephson junction quality factor of *Q* ∼ 10^5^, the relaxation time is *t*_r_ ∼ 1 μs. From this rough estimate it can be seen that in the future, adiabatic cells of tuning circuits can also be used as auxiliary qubits for a more efficient use of structures on a “quantum” chip.

## Results and Discussion

We have already considered the system presented in the article in our previous work [30]. But we considered it exclusively as a classical adiabatic neural network cell, even when we studied its dynamics in the quantum mode. The main result of this work is to demonstrate a good quantum logic operation with a relatively short duration on a single-contact inductively shunted interferometer (see Figure 2). After all, such a system has not been considered commonly by the modern scientific community to be a good flux qubit. We were also surprised by the possibility of controlling the qubit state using unipolar current pulses, which greatly simplifies all control electronics [51–52]. But won’t such a deliberately non-resonant (broadband) impact lead to a parasitic leakage of the state to higher levels? We have conducted a separate study of this issue, the results of which are presented in this section.

**Figure 5:**
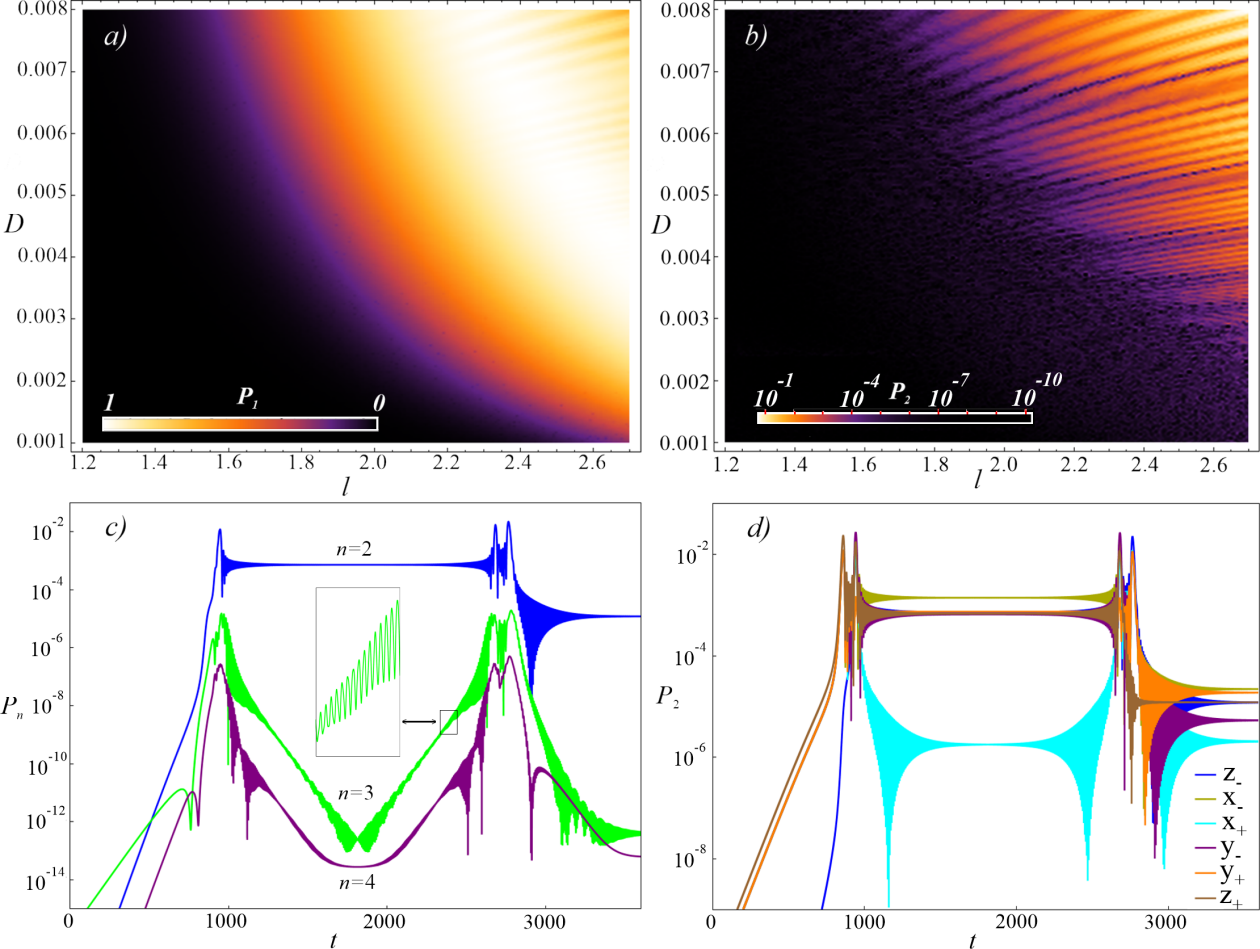
Interference population map for the first excited state (a) and the second state (b) for different values of the inductance *l* and rates of change of the control field fronts *D*. (c) Time-dependent dynamics of the overlying states (*n* = 2, 3, 4) and (d) leakage to the second excited level (*n* = 2) for the system initialised at different poles on the Bloch sphere for the values *l* = 2.63 and *D* = 0.0044, similar to those shown in Figure 2a. The parameters of the system were *l*_a_ = 1 + *l*, *l*_out_ = 0.1, *E*_J_ = 2*E*_c_, and *t*_1_ = 3*t*_2_.

The amount of leakage from the computational qubit basis when implementing a good “NOT” operation is mainly influenced by (i) the distance between the converging energy levels, Δ*t* = *t*_2_ − *t*_1_, and (ii) the rise/fall rate of the control pulse *D*_R,F_. At the same time, a fairly significant range of acceptable parameters is highlighted (the light colour in Figure 5a) when the “NOT” operation is implemented in the system via Landau–Zener transitions. Usually, this “working” range of parameters varies from *l* = 2.2 for *D* = 0.008 up to *l* = 2.7 for *D* = 0.0035, which gives an estimated duration of the operation of 20–60 ns (for the selected typical parameters of the Josephson contact, *I*_c_ = 50 nA and *C* = 6 fF). Furthermore, in order to assess the quality of this operation and to control the leakage into the overlying states in Figure 5b, we show the behaviour of the population of the second excited level, *P*_2_, in the system for various values of *D* and *l*. The leakage into the second state is dominant (see Figure 5c). The oscillations on the curves are due to a more complex double anticrossing (in the case of leakage to *P*_2_, the blue curve in Figure 5c, this is the convergence of the first and second levels) and the acquired progression of Stuckelberg phases. Similar behaviour was also found for different initial states of the qubit, Figure 5d. We have evaluated the reliability of such an operation based on the calculation of fidelity. We evaluate the fidelity of the gate *U*_g_ (for Figure 2a) as in [53]:


[20]

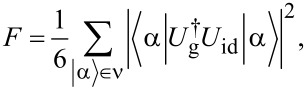



where the summation runs over the six states ν aligned along the cardinal directions of the Bloch sphere 
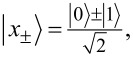
 
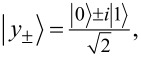
 |*z*_+_⟩ = |0⟩, |*z*_−_⟩ = |1⟩. Here, |0⟩ and |1⟩ are the ground and first excited states of the system, and *U*_id_ is the matrix of an ideal qubit gate. For the “NOT” operation shown in Figure 2a for *l* = 2.63 and *D* = 0.0044, taking into account the optimisation of the pulse parameters Δ*t*, according to Figure 2g and Figure 5, we can get the fidelity of the operation *F* = 99.99%. We can therefore say with confidence that the same cell can be used both as a classical adiabatic neuron and as a qubit whose state can be controlled with an infidelity of the order of 0.0001.

## Conclusion

The simplest cell of adiabatic superconducting logic can function even in quantum mode as an element of tuning circuits if the control signals change quasi-adiabatically with time (rise/fall times for control fields are more than a 100 ns). At sufficiently low temperatures and relatively small normalised inductances, such an inductively shunted single-contact interferometer can also be used as part of a perceptron-type neural network to process signals received from qubits. Such a cell can be used in quantum mode also as an auxiliary qubit with relatively fast “flux” control. Future research will address the problem of using more advanced adiabatic superconducting logic cells for such purposes. In addition, bifunctional cells, which can act as adiabatic neurons or flux qubits depending on the operating conditions, have the potential to be used to simulate the operations in a non-classical brain [54–55].
